# Dehydrobufotenin extracted from the Amazonian toad *Rhinella marina* (Anura: Bufonidae) as a prototype molecule for the development of antiplasmodial drugs

**DOI:** 10.1590/1678-9199-JVATITD-2020-0073

**Published:** 2021-01-08

**Authors:** Felipe Finger Banfi, Gabriela Camila Krombauer, Amanda Luisa da Fonseca, Renata Rachide Nunes, Silmara Nunes Andrade, Millena Alves de Rezende, Mariana Helena Chaves, Evaldo dos Santos Monção, Alex Guterres Taranto, Domingos de Jesus Rodrigues, Gerardo Magela Vieira, Whocely Victor de Castro, Fernando de Pilla Varotti, Bruno Antonio Marinho Sanchez

**Affiliations:** 1 Laboratory of Immunopathology and Tropical Diseases, Health Education and Research Center (NUPADS), Institute of Health Sciences, Federal University of Mato Grosso, Sinop, MT, Brazil.; 2 Research Center on Biological Chemistry (NQBio), Federal University of São João Del Rei, Divinópolis, MG, Brazil.; 3 Department of Chemistry, Federal University of Piauí, Teresina, PI, Brazil.; 4 Center for Biodiversity Studies in the Amazon Region of Mato Grosso (NEBAM), Federal University of Mato Grosso, MT, Brazil.; 5 Quality Control Laboratory, Federal University of São João Del Rei, Divinópolis, MG, Brazil.

**Keywords:** Bufadienolides, Antimalarial drug Docking, Natural compounds

## Abstract

**Background:**

The resistance against antimalarial drugs represents a global challenge in the fight and control of malaria. The Brazilian biodiversity can be an important tool for research and development of new medicinal products. In this context, toxinology is a multidisciplinary approach on the development of new drugs, including the isolation, purification, and evaluation of the pharmacological activities of natural toxins. The present study aimed to evaluate the cytotoxicity, as well as the antimalarial activity *in silico* and *in vitro* of four compounds isolated from *Rhinella marina* venom as potential oral drug prototypes.

**Methods:**

Four compounds were challenged against 35 target proteins from *P. falciparum* and screened to evaluate their physicochemical properties using docking assay in Brazilian Malaria Molecular Targets (BraMMT) software and *in silico* assay in OCTOPUS® software. The *in vitro* antimalarial activity of the compounds against the 3D7 *Plasmodium falciparum* clones were assessed using the SYBR Green I based assay (IC_50_). For the cytotoxic tests, the LD_50_ was determined in human pulmonary fibroblast cell line using the [3(4,5-dimethylthiazol-2-yl)-2,5-diphenyltetrazolium bromide] (MTT) assay.

**Results:**

All compounds presented a ligand-receptor interaction with ten *Plasmodium falciparum*-related protein targets, as well as antimalarial activity against chloroquine resistant strain (IC_50_ = 3.44 μM to 19.11 μM). Three of them (dehydrobufotenine, marinobufagin, and bufalin) showed adequate conditions for oral drug prototypes, with satisfactory prediction of absorption, permeability, and absence of toxicity. In the cell viability assay, only dehydrobufotenin was selective for the parasite.

**Conclusions:**

Dehydrobufotenin revealed to be a potential oral drug prototype presenting adequate antimalarial activity and absence of cytotoxicity, therefore should be subjected to further studies.

## Background

Malaria is an important human parasitic disease, occurring in tropical and subtropical areas of the planet [1]. The malaria parasites resistance to ancient antimalarials consists of the biggest hurdles to malaria control [2]. Because of the resistance to antimalarials, artemisinin and its derivatives have been the first-line antimalarial agents against *Plasmodium falciparum* [3,4]. Artemisinin-based combination therapies (ACT) are the most effective regimens for the first-line treatment for *P. falciparum* infections. Despite of the WHO recommendations for using and prescribing the ACTs, pharmacokinetic and pharmacodynamic studies with *P. falciparum* strains have already demonstrated the development of resistance to these compounds [5,6,7]. This phenomenon is responsible to increase the mortality in endemic areas contributing to the appearance and expansion of new outbreaks of *P. falciparum* malaria. Thus, new strategies are required to prevent increased resistance to ACTs. In addition, a potential strategy would be to add a third drug with independent antiparasitic activity [6]. 

Natural products have providing a great contribution to the development of new drugs [8]. In fact, many of the antimalarial drugs commercially available are derivatives of phytoconstituents [9]. In addition to the plant-derived remedies, animal extracts, products, and even secretions are also a source of a plethora of therapeutical agents [10]. 

The venoms secreted by the paratoid glands of amphibians from the order Anura is the first line of defense against predators and microorganisms [11,12]. The toads of *Bufonidae* family have been widely studied due to the bioactive properties found in the *Rhinella marina* venom, which have already shown antitumor [13,14,15], antiviral [16,17], and antiparasitic activities [18]. The cholesterol-derived steroid structures called bufadienolides are major active compounds in the venom of *Bufonidae* family and are considered a promising source of bioproducts [19,20]. Furthermore, the alkaloids dehydrobufotenin and bufotenine also identified in *R. marina* venom have demonstrated to possess antiproliferative and antiviral activity, respectively [20,21, 22, 23].

The development of malaria drugs is slower than that involving the antibacterial drugs [24]. However, this process can be speeded up with the aid of computational drug planning tools, known as molecular modeling or *docking*, to design new compounds and to study their respective protein targets [25, 26]. The *docking* is a robust tool for investigating the chemical interactions of ligands and receptors and to explore the structural factors related to the biological effect [27, 28]. 

To date, there are almost no studies investigating compounds isolated from the bufonides venom as potential new antimalarial drugs. Therefore, present study aimed to evaluate the cytotoxicity, as well as the antimalarial activity *in silico* and *in vitro* of four compounds isolated from *Rhinella marina* venom as potential oral drug prototypes.

## Methods

### Sample collection

The animals (*R. marina*) were collected in the Branca de Neve Community, Mato Grosso, Brazil (Latitude 11°51'51.59 "S/Longitude 55°22'47.99" W), from January to March of 2015, in the municipality of Sinop, Mato Grosso state, North-Western Brazil. The vegetation where the individuals were found is classified as dense humid forest and the climate of the region is tropical with an average temperature of 24° C, relative air humidity of ~80%, and average annual rainfall of 2,034 mm. 

The amphibians were captured and identified by the biologist (D. J. Rodrigues - 95 IBAMA, SISBIO: number 30034-1). The secretions were obtained by manual compression of the parotoid macrogland and the animals were returned to nature after this procedure. The voucher specimens (*R. marina* - ABAM-H 2256) were collected and deposited in the zoological collection (Acervo Biológico da Amazônia Meridional) of the Federal University of Mato Grosso located at Sinop city (the collection permit was issued by the Chico Mendes Institute for Biodiversity Conservation).

All experiments were performed according to internationally accepted guidelines for the care and use of laboratory animals and were previously approved by the Federal University of Mato Grosso Institutional Animal Care and Use Committee (Protocol 23108.700260/14-7) and National System for the Management of Genetic Heritage and Associated Traditional Knowledge (SisGen AE 19081).

### **Extraction of *R. marina* Venom samples and isolation**

**Figure 1.**
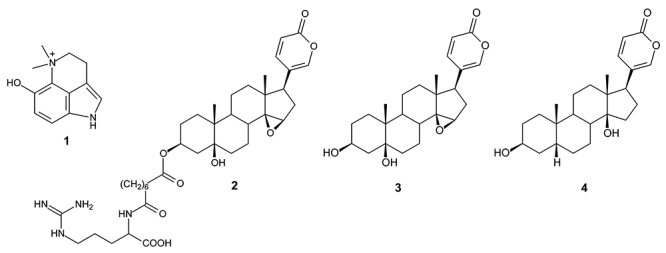
Molecular structures of *R. marina* venom fractions. **(1)** Dehydrobufotenine (CRV - 28), **(2)** marinobufotoxin (CRV-6-21-58), **(3)** marinobufagin (MB-1) and **(4)** bufalin (MB-3).

*R. marina* toad venoms were dried, powdered and extracted three times (3 x 20 mL) with 100% methanol (MeOH) in ultrasound waves for 10 minutes at room temperature [14]. The extract was fractionated on Sephadex LH-20 column using methanol as eluent. Four fractions were obtained: CRV-6 (783.8 mg); CRV-28 (102.9 mg); CRV-52 (315.8 mg) and CRV-70 (394.1 mg). The structure of the isolated compounds marinobufotoxin, dehydrobufotenin, marinobufagin, and bufalin are presented in Figure 1 [29]. 

### Evaluation of molecular docking

The compounds were designed using MarvinSketch^®^ software (ChemAxon, Cambridge, MA, USA) and the molecular structures were refined through MOPAC® software (Stewart Computational Chemistry, Colorado Springs, CO, USA) using the PM7 semi-empirical method. The compounds dehydrobufotenine, marinobufotoxin, marinobufagin and bufalin were submitted to the molecular docking calculations in the AutoDock Vina® program [30] using OCTOPUS^®^ platform [31] and the configuration files were determined through a re-docking step [32]. Thus, the virtual screening of antimalarial drugs was performed using the BraMMT data bank according [33]. From the generated binding energy values, Δ (binding energy of the crystallographic ligand - binding energy of the compound) values were calculated. Thus, Δ values greater than “0”, show that it has higher binding energy than the crystallographic ligand, suggesting greater interaction with the target. Table 1 lists the molecular targets used to build the BraMMT platform.

**Table 1.** Molecular targets, location and enzymatic class of the 35 molecular targets obtained from the Tropical Disease Research (TDR target database) for building the Brazilian Malaria Molecular Targets (BraMMT).

PDB Code	Name	Enzymatic class	Location
**1LF3**	Plasmepsin II	Hydrolase	Digestive vacuole
**1LYX**	Triosephosphate Isomerase (PfTIM)-Phosphoglycolate	Isomerase	Cytoplasm
**1NHW**	Enoyl-acyl-carrier-protein reductase	Oxidoreductase	Apicoplast
**1O5X**	Triosephosphate Isomerase	Isomerase	Cytoplasm
**1QNG**	Peptidyl-prolil cis-trans isomerase	Isomerase	Cytoplasm
**1RL4**	Formylmethionine deformylase	Hydrolase	Apicoplast
**1TV5**	Dihydroorotate dehydrogenase	Oxidoreductase	Cytoplasm e Nucleus
**1U4O**	L-lactate dehydrogenase	Oxidoreductase	Cytoplasm
**1YWG**	glyceraldehyde-3-phosphate dehydrogenase	Oxidoreductase	Cytoplasm
**2AAW**	Glutathione s-transferase	Transferase	Cytoplasm
**2ANL**	Plasmepsin IV	Hidrolase	Digestive vacuole
**2OK8**	Putative ferredoxin--NADP reductase	Oxidoreductase	Apicoplast
**2PML**	Ser/Thr protein kinase	Transferase	Cytoplasm
**2Q8Z**	Orotidine-monophosphate-descarboxylase	Liase	Nucleus
**2VFA**	Hypoxantine-guanine phosphoribosyltransferase	Transferase	Apicoplast
**2VN1**	70 KDA peptidylprolyl isomerase	Isomerase	Nucleus
**2YOG**	Thymidylate kinase	Transferase	Nucleus
**3AZB**	Beta-hydroxyacyl-ACP dehydratase	Lyase	Cytoplasm
**3BPF**	Falcipain II	Hydrolase	Digestive vacuole
**3CLV**	Rab5 Protein	Signaling protein	Cytoplasm
**3FNU**	HAP Protein	Hydrolase	Digestive vacuole
**3K7Y**	Aspartate aminotransferase	Transferase	Cytoplasm
**3N3M**	Orotidine 5’-phosphate decarboxylase	Lyase	Apicoplast
**3PHC**	Purine nucleoside phosphorylase	Transferase	Nucleus
**3QS1**	Plasmepsin I	Hydrolase	Digestive vacuole
**3T64**	Deoxyuridine 5’-triphosphate nucleotidohydrolase	Hydrolase	Nucleus
**3TLX**	Adenylate kinase 2	Transferase	Cytoplasm and mitochondria
**4B1B**	Thioredoxin reductase	Oxidoreductase	Cytoplasm
**4C81**	22-C-Methyl-D-Erythritol 2,4-Cyclodiphosphate synthase	Lyase	Apicoplast
**4J56**	Thioredoxin reductase 2	Oxidoreductase	Cytoplasm
**4N0Z**	Glutaredoxin	Oxidoreductase	Cytoplasm
**4P7S**	Macrophage migration inhibitory factor-like protein	Cytokine inhibitor	Cytoplasm
**4QOX**	Calcium-dependent protein kinase 4	Transferase	Cytoplasm
**PfATP6**	Calcium pump ortholog ATPase	Transporter	Membrane
**PfHT** (10.5452/ma-aej21)	Hexose carrier protein	Transporter	Membrane

### Evaluation of physicochemical and ADMET properties

The physicochemical and ADMET properties of the compounds dehydrobufotenin, marinobufotoxin, marinobufagin and bufalin (CRV-28, CRV-6-28-51, MB-1 and MB-3, respectively) were analyzed using DataWarrior^®^ software and SwissADME website [34]. The properties of molecular mass, partition coefficient (ClogP), number of hydrogen donor groups, and number of hydrogen acceptor groups were predicted. The toxicological characteristics of the ligand, such as mutagenicity, tumogenicity, and irritability, were analyzed [35]. Finally, the pharmacokinetic processes of absorption, distribution, metabolism, excretion, and toxicity were estimated [34].

### ***In vitro* culture of *P. falciparum***

*P. falciparum* W2 strain (chloroquine resistant) [36, 37, 38] was cultured in blood stage culture to test the antiplasmodial efficacy of toad venom compounds (1, 2, 3, and 4). *P. falciparum* continuous culture was maintained as previously described [36,39] with minor modifications. Parasites were maintained at 5% hematocrit using type O^+^ human erythrocytes in RPMI 1640 medium (Sigma-Aldrich^®^, St. Louis, Missouri, USA) supplemented with 25 mM NaHCO_3_, 1.0% albumax, 45 mg/L hypoxanthine, 40 μg/mL gentamycin and incubated at 37º C under approximately 5% of CO_2_. The parasites at early stages were synchronized at ring stage by sorbitol treatment [40]. Initial parasitemia was adjusted to 0.5% with 2% hematocrit in all experiments.

### ***In vitro* antiplasmodial activity**

*In vitro* antiplasmodial activity of the bufadienolides (compounds dehydrobufotenin, marinobufotoxin, marinobufagin and bufalin) was done in 96 well plates [41]. The growth inhibition of intraerythrocytic forms and parasite morphology in culture was assessed by microscopic observation of the Giemsa-stained thin blood films. Ring stage parasites (0.5% parasitemia and 2% hematocrit) were added to each well of 96-well microculture plates. The compounds (dehydrobufotenin, marinobufotoxin, marinobufagin and bufalin) were dissolved in DMSO and diluted to concentrations ranging from 0.78 to 100 μg/mL using complete medium and stored a 4º C. After incubation at 37º C for 48 hours, *P. falciparum* growth inhibition was assessed in Giemsa-stained smears by observing 5,000 erythrocytes per 1 thin blood film in triplicate. The culture medium was replaced with fresh medium with or without test samples/control drugs. Chloroquine (CQ) was used as a reference antimalarial. The activity of the compounds (dehydrobufotenin, marinobufotoxin, marinobufagin and bufalin) was expressed as the percentage reduction in parasitemia relative to controls without drugs. All experiments were performed in triplicate. The results were expressed as the mean of the IC_50_ (Drug concentration that reduced parasite viability in 50%).

### ***In vitro* cytotoxicity**

*In vitro* cytotoxicity of each compound was assessed on WI-26VA4 (ATCC CCL-95.1, USA) human pulmonary fibroblast cells. The cells were cultured in RPMI-1640 (Sigma-Aldrich^®^, St. Louis, Missouri, USA) medium supplemented with 10% heat-inactivated fetal bovine serum and 100 μg/mL of gentamycin in a 5% CO_2_ atmosphere at 37º C. The cells were washed with culture medium, trypsinized, distributed in a flat-bottomed 96-well plate (5×10^3^ cells/well), and incubated for 18 hours at 37º C for cell adherence [42]. The compounds (20 μL) were diluted in different concentrations ranging from 0.2 - 200 μg/mL and incubated with the cells for 24 hours in a 5% CO_2_ atmosphere at 37º C.

A 3-(4,5-dimethylthiazol-2-yl)-2,5-diphenyltetrazolium bromide (MTT) solution (5 mg/mL; 20 μL/well) was added to evaluate mitochondrial viability; after a further 3 hours incubation, the supernatants were carefully removed, 100 μL of DMSO was added to each well, and the reactions were mixed to solubilize the formazan crystals. The optical density was determined at 540 nm to measure the signal and background, respectively (Spectra Max340PC^384^, Molecular Devices, Sunnyvale, California, USA) [43, 44, 45, 46, 47, 48]. The cell viability was expressed as a percentage of the control absorbance in the untreated cells after subtracting the appropriate background.

The minimum lethal dose for 50% of the cells (LD_50_) was determined as described [49].

### Selectivity index (SI)

A selectivity index (SI) corresponding to the ratio between the cytotoxic and antiplasmodial activities of each compound tested. The values greater than 10 were considered indicative of lack of toxicity, whereas the substances with values below 10 were considered toxic [38]. The SI index was calculated as follow:

$$SI=\frac{{LD}_{50}\;Cell}{{IC}_{50}\;P.falciparum}$$

### Statistical analysis

The concentrations of compounds able to inhibit 50% of parasite growth (IC_50_) were determined based on the equation of the curve obtained by plotting the % of parasitemia regression vs the log of the concentration of compound. The coefficients of regression of these curves were calculated using the method of least squares. The LD_50_ were determined based on the equation of the curve obtained by plotting the % of cellular death versus the concentration of compound (GraphPad Prism Software, version 5.0 for Windows, San Diego, California, USA). The average IC_50_ and LD_50_ were compared using ANOVA. Statistical significance was defined at the 5% level (P<0.05).

## Results

### Compounds

***Compound 1 (CRV - 28)***

Dehydrobufotenin - molecular formula: C_15_H_12_N_2_O; IT-ESI-MS [M+H]^+^ 203.1; ^1^H NMR (CD_3_OD- 600 MHz): *δ* 7.11(s, 1H), *δ* 6.81(d, *J* = 8.6 Hz, 1H), *δ* 7.29(d, *J* = 8.7 Hz, 1H), *δ* 3.29(d, *J* = 5.8 Hz, 2H), *δ* 4.1(t, *J* = 5.9 Hz, 2H) and *δ* 3.68(s, 6H). ^13^C NMR (CD_3_OD - 150 MHz): *δ* 122.5, *δ* 120.6, *δ* 104.6, *δ* 121.1, *δ* 149.0, *δ* 115.0, *δ* 118.9, *δ* 128.9, *δ* 20.0, *δ* 69.6 and *δ* 54.0.

The CRV-6 fraction was submitted to the Sephadex LH-20 column with MeOH. The sub-fractions CRV-6-28 was further fractionated by silica gel column, eluted in CHCl_3_/MeOH with an increasing polarity gradient system. The subgroup obtained was CRV-6-28-51 (35.1 mg) and through NMR analysis and mass spectrometry was identified as marinobufotoxin (2).

***Compound 2 (CRV-6-28-51)***

 3-(N-suberoylargininyl) marinobufagin (marinobufotoxin) - molecular formula: C_38_H_56_N_4_O_9_; IT-ESI-MS [M+H]^+^ 713.5; ^1^H NMR (CD_3_OD - 600 MHz): *δ* 5.14 (m), *δ* 3.68 (s, 1H), *δ* 2.56 (d, *J*= 9.9 Hz, 1H), *δ* 0.73 (s, 3H), *δ* 0.94 (s, 3H), *δ* 7.46 (d, *J* = 1.8 Hz, 1H), *δ* 7.90 (dd, *J* = 9.8 and 2.4 Hz, 1H), *δ* 6.28 (t, *J* = 9.6 Hz, 1H), *δ* 4.28 (dd, *J* = 8.4 and 4.9 Hz, 1H), *δ* 1.88 (m, 2H), *δ* 1.61 (m, 2H), *δ* 3.20 (m, 2H), *δ* 2.20-2.38 (m, 4H), *δ* 1.71 (m, 4H) and *δ* 1.36 (m, 4H). ^13^C NMR (CD_3_OD - 150 MHz) δ (ppm): 26.4, 25.7, 72.2, 36.4, 74.3, 36.1, 24.3, 33.9, 43.2, 41.6, 22.5, 39.7, 46.1, 75.7, 61.1, 27.7, 48.3, 17.0, 17.1, 124.5, 150.6, 149.7, 115.4, 164.6, 55.2, 31.1, 25.8, 42.0, 174.8/175.7, 35.4/37.3, 26.3/26.6, 29.4/30.0, 178.8 and 158.6.

The CRV-70 fraction (394.1 mg) was fractionated in silica gel column. The CRV-70-04 sub-fraction was analyzed by NMR and mass spectrometry, and its majority compound was identified as marinobufagin (**3**). Subsequently, this sub-fraction was submitted to purification by High Performance Liquid Chromatography (HPLC) using ultrapure water (eluent A) and acetonitrile (eluent B), the system was eluted in isocratic mode with 60% eluent B, obtaining the group MB-3 (7.9 mg), which was identified as bufalin (**4**). The spectral data of the isolated compounds are in accordance with the literature [25, 46, 47, 48] and described below. The structures are shown in Figure 1.

***Compound 3 (MB-1)***

Marinobufagin - molecular formula: C_24_H_32_O_5_; IT-ESI-MS [M+H]^+^ 401.3; ^1^H NMR (CDCl_3_ - 600 MHz): *δ* 4.16-4.19 (m), *δ* 3.49 (s, 1H), *δ* 2.46 (d, *J*= 10.1Hz, 1H), *δ* 0.77 (s, 3H), *δ* 0.97 (s, 3H), *δ* 7.23 (d, *J* = 9.8 Hz, 1H), *δ* 7.76 (dd, *J* = 9.8 and 2.5 Hz, 1H) and *δ* 6.24 (dd, *J* = 9.8 and 0.8 Hz, 1H). ^13^C NMR (CDCl_3_ - 150 MHz): 24.9, 28.1, 68.1, 39.5, 74.7, 34.8, 23.4, 32.7, 42.8, 41.0, 21.6, 39.5, 45.2, 74.7, 59.9, 32.4, 47.7, 16.9, 16.9, 122.4, 149.8, 147.0, 115.4 and 162.2.

***Compound 4 (MB-3)***

 Bufalin - molecular formula: C_24_H_34_O_4_; IT-ESI-MS [M+H]^+^ 387.3; ^1^H NMR (CDCl_3_ - 600 MHz): *δ* 4.13-4.18 (m), *δ* 2.56 (dd, *J* = 9.7 and 6.6 Hz, 1H), *δ* 0.69 (s, 3H), *δ* 0.94 (s, 3H), *δ* 7.22 (d, *J* = 1.8 Hz, 1H), *δ* 7.84 (dd, *J* = 9.7 and 2.6 Hz, 1H) and *δ* 6.26 (d, *J* = 9.7 Hz, 1H) ^13^C NMR (CDCl_3_ - 150 MHz): 29.8, 28.0, 66.9, 33.4, 36.1, 26.6, 21.5, 42.5, 35.8, 35.5, 21.5, 41.0, 48.5, 85.5, 32.8, 28.8, 51.3, 16.7, 23.9, 122.8, 148.6, 146.9, 115.5 and 162.6. 

### ***In silico*: Virtual screening**

Table 1 shows the molecular targets, location, and enzymatic class of the 35 molecular targets obtained from the Tropical Disease Research (TDR targets database) for building the Brazilian Malaria Molecular Targets (BraMMT). The compounds (dehydrobufotenin, marinobufotoxin, marinobufagin, bufalin) were assayed for the docking methodology in the BraMMT data bank. Virtual screening performed against the 35 molecular targets in the database using OCTOPUS software presented 10 potential targets for all compounds tested (Table 2). These results were found when the binding energy values are lower than the crystallographic control.

**Table 2.** Binding energies between compounds tested and molecular targets (kcal.mol^-1^).

Compounds	Binding energy (kcal.mol^-1^)																			
	1NHW	Δ	1O5X	Δ	2OK8	Δ	2VFA	Δ	3AZB	Δ	3BPF	Δ	4N0Z	Δ	4P7S	Δ	PfATP6	Δ	PfHT	Δ
**Crystallographic**	-8.3		-5.3		-2.0		-5.8		-6.3		-6.3		-4.3		-6.0		-7.2		-5.7	
**Dehydrobufotenine**	-6.9	-1.4	-5.6	0.3	-2.6	0.6	-2.1	-3.7	-0.4	-5.9	-5.8	-0.5	-3.9	-0.4	-5.0	-1.0	-6.6	-0.6	-5.8	0.1
**Marinobufotoxin**	-9.9	1.6	-5.9	0.6	-7.9	5.9	-9.2	3.4	-6.8	0.5	-7.5	1.2	-6.6	2.3	-7.3	1.3	-9.3	2.1	-8.8	3.1
**Marinobufagin**	-10.9	2.6	-5.8	0.5	-8.4	6.4	-9.0	3.2	-6.6	0.3	-7.7	1.4	-6.6	2.3	-7.9	1.9	-8.2	1.0	-9.9	4.2
**Bufalin**	-10.8	2.5	-6.1	0.8	-7.9	5.9	-9.4	3.6	-7.2	0.9	-7.1	0.8	-7.1	2.8	-8.6	2.6	-8.3	1.1	-9.3	3.6
***D*-glucose**	-		-		-		-		-		-		-		-		-		-4.3	0.2

**Figure 2.**
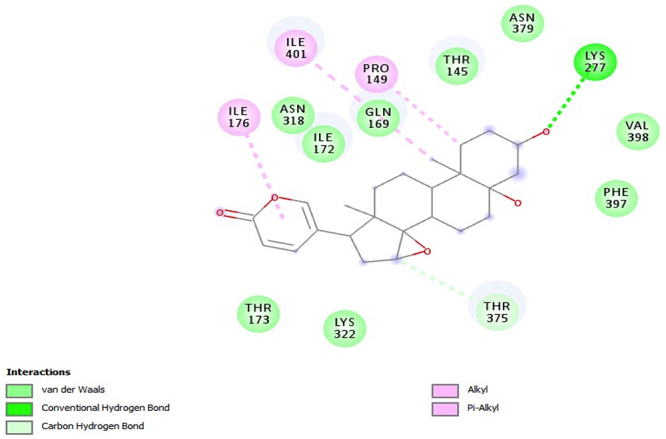
Residues in the active site of PfHT target interacting with the compound marinobufagin (MB-1).

**Figure 3.**
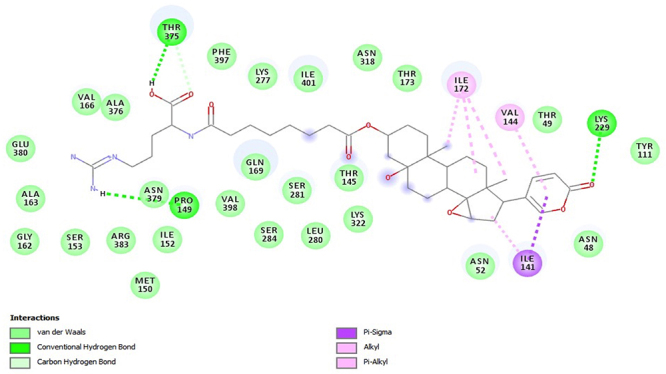
Residues in the active site of PfHT target interacting with compound the marinobufotoxin (CRV-6-21-58).

**Figure 4.**
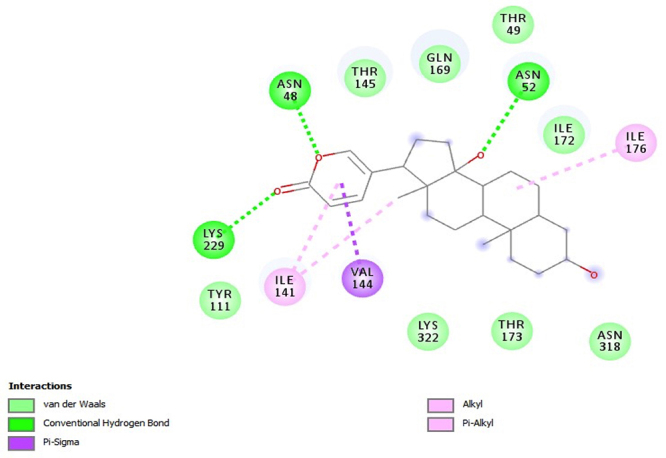
Intermolecular interactions of the compound bufalin (MB-3) with PfHT.

**Figure 5.**
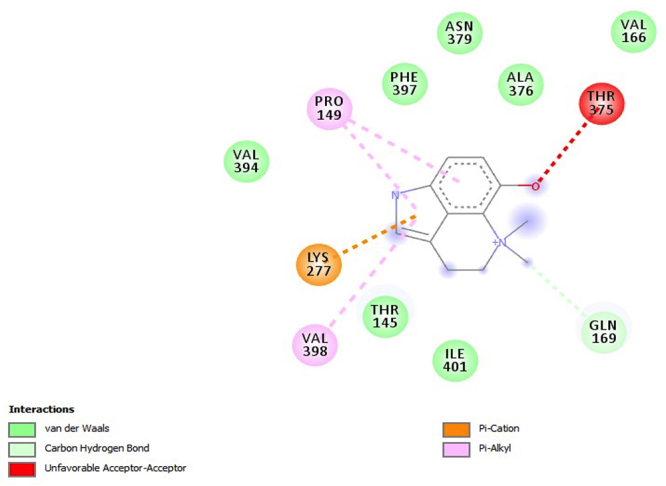
Intermolecular interactions of the compound dehydrobufotenine (CRV-28) with PfHT.

**Figure 6.**
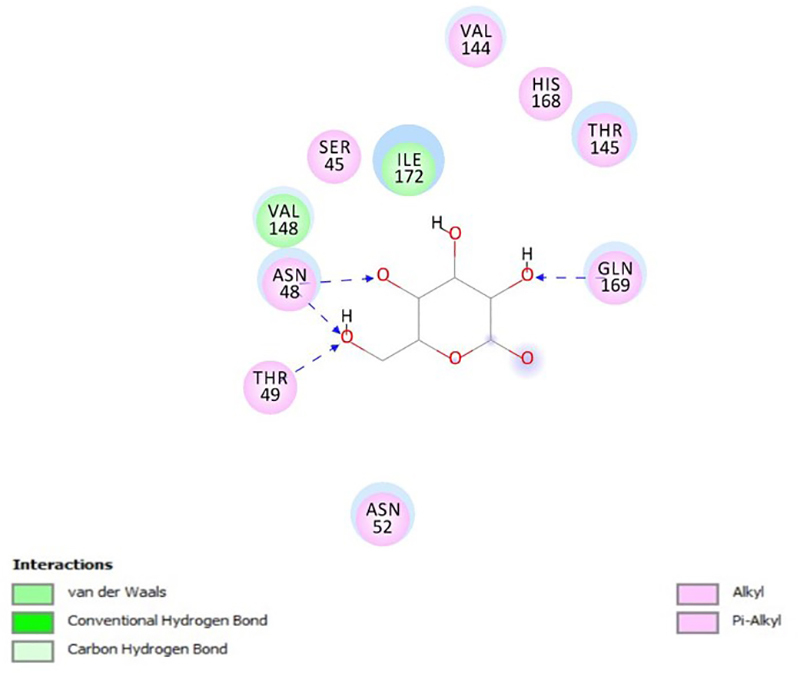
Intermolecular interactions of *D*-glucose with PfHT.

**Figure 7.**
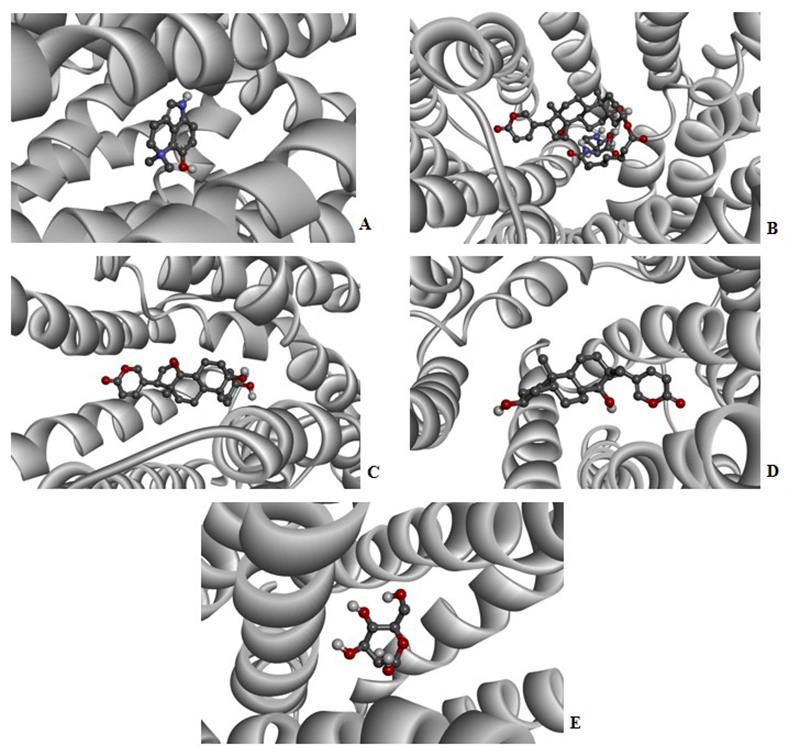
Three-dimensional structure of PfHT complexed with of *R. marina* venom fractions. **(A)** Dehydrobufotenine (CRV-28), **(B)** marinobufotoxin (CRV-6-21-58), **(C)** marinobufagin (MB-1), **(D)** bufalin (MB-3) and **(E)** *D*-glucose.

PfHT is characterized as glucose transporter of *P. falciparum*. The docking, QM/MM and molecular dynamics simulations were already performed by our group [49, 50, 51]. The Figures 2, 3, 4, 5 and 6 shows 2D ligand-receptor interactions maps with PfHT. The figures show the chemicals bonds that occurred between the compound and the target, which enables the identification of pharmacophoric groups and possible structural improvements for better oral permeability, absorption, and bioavailability. The 3D binding diagram of the compounds is show in Figure 7. Docking with *D*-Glucose and PfHT was performed as a control.

Marinobufagin binds to 2-pyrone group in PfHT protein by residue ILE-176 while the perhydrophenanthrene nucleus binds by residues ILE-401 and PRO-149 (Figure 2). Marinobufotoxin binds to 2-pyrone group by residue VAL-144 while the perhydrophenanthrene nucleus binds by residues ILE-172 (Figure 3). Bufalin interacts with the protein electrostatically from the same active sites with residues ILE-141 and ILE-176 (Figure 4).

The compound dehydrobufotenin is an alkaloid derivative with consequent presence of the quinoline ring like the quinine and its analogs. Among the interactions involving the binding to the PfHT protein, the quinoline nucleus performs electrostatic bonding with the PRO149 residue (Figure 5). Although there is an unfavorable interaction between compound dehydrobufotenin and PfHT, this negative event is offset by other interactions. 

*D*-glucose interacts in the same biding site of PfHT with electrostatic bonding in the residues GLN169 and THR145 (Figure 6).

Table 3 presents the information obtained from the physical-chemical properties by the DataWarrior^®^ software. The compound marinobufotoxin presented CLogP < 5 (2.80), molecular mass was greater than 500 (712.88 g/mol), the hydrogen accepting groups that performed interactions are more than 10 (13 groups), and the hydrogen donor groups are more than 5 (6 groups). Thus, based on the rules of Lipinski, compound marinobufotoxin is expected to present unfavorable pharmacokinetics properties (absorption, distribution, metabolization, excretion, and toxicity).

**Table 3.** Molecular mass (g/mol), partition coefficient (CLogP), number of hydrogen donor groups and number of hydrogen acceptor groups of tested compounds.

Compounds	Molecular mass	CLogP	H^+^ Acceptors	H^+^ Donors
**Dehydrobufotenine**	203.26	0.51	3	2
**Marinobufotoxin**	712.88	2.80	13	6
**Marinobufagin**	400.51	1.87	5	2
**Bufalin**	386.53	2.99	4	2

The compounds dehydrobufotenin, marinobufagin and bufalin presented molecular mass below 500 (203.26 g/mol; 400.51 g/mol and 383.53 g/mol, respectively), CLogP <5 (0.51; 1.87 and 2.99, respectively), less than 10 hydrogen acceptor groups (3 groups; 5 groups and 4 groups, respectively) and less than 5 hydrogen donor groups (2 groups). Thus, these results, based on the rules of Lipinski, the compounds dehydrobufotenin, marinobufagin and bufalin have sufficiently acceptable ADMET properties. 

Gleeson [52] suggests in his study that compounds with CLogP less than 4 and molecular weight less than 400 have a more favorable ADMET profile than those suggested by Lipinski. Following the Gleeson theory, all compounds have CLogP < 3 and compounds dehydrobufotenin, marinobufagin and bufalin have molecular mass below 400. 

Toxicological characteristics of the four compounds using the DataWarrior^®^ software, factors such as mutagenicity, tumogenicity or irritability were not evidenced (Table 4).

**Table 4.** Toxicological characteristics of compounds obtained from *R. marina* venom.

Compounds	Mutagenicity	Tumorgenicity	Irritability
**Dehydrobufotenine**	Absent	Absent	Absent
**Marinobufotoxin**	Absent	Absent	Absent
**Marinobufagin**	Absent	Absent	Absent
**Bufalin**	Absent	Absent	Absent

### ***In vitro* antiplasmodial activity**

The compounds dehydrobufotenin, marinobufotoxin, marinobufagin and bufalin diluted in DMSO were assayed for antiplasmodial activity against chloroquine-resistant *P. falciparum* W2. Table 5 shows the antiplasmodial activity of dehydrobufotenin, marinobufotoxin, marinobufagin and bufalin in two different experiments. Starting from 100 μg/mL, the compounds were diluted to various concentrations (0.78-100 μg/mL) to calculate the IC_50_ values. The samples (dehydrobufotenin, marinobufotoxin, marinobufagin and bufalin) showed IC_50_ values ranged from 3.44 to 19.11 μM (Table 5). The marinobufagin and bufalin had the IC_50_ values close to chloroquine, the antimalarial used as a positive control.

**Table 5.** The lethal drug concentration that reduced parasite viability in 50% (IC_50_), lethal drug concentration that reduced WI-26VA4 cells viability in 50% (LD_50_), and selectivity index (SI) values obtained from *in vitro* tests with venom fractions from *R. marina* venom, and chloroquine (CQ) against *P. falciparum* W2 strain.

Compounds	IC_50_ ± SD (μM)*	LD_50_^a^ ± SD (μM)*	SI^a^
**Dehydrobufotenine**	19.11 ± 0.20	235.76 ± 4.03	12.33
**Marinobufotoxin**	5.31 ± 0.25	8.89 ± 2.66	1.67
**Marinobufagin**	3.89 ± 0.42	3.04 ± 0.25	0.78
**Bufalin**	3.44 ± 0.43	25.9 ± 7.04	7.52
**Chloroquine (CQ)**	1.04 ± 0.21	>100	>100

^a^LD_50_ and SI values were obtained with MTT cytotoxic test in human pulmonary fibroblast cells (WI-26VA4).

*Mean and standard deviation (SD) of triplicate experiments.

### Cytotoxic activity on human pulmonary fibroblast cells

To evaluate the cytotoxic activity of the compounds dehydrobufotenin, marinobufotoxin, marinobufagin and bufalin, the MTT assay conducted in human pulmonary fibroblast cells (WI-26VA4). It was observed that the compounds marinobufotoxin, marinobufagin and bufalin showed high cytotoxicity to this cell line with low LD_50_ values (marinobufotoxin = 8.89 μM; marinobufagin = 3.04 μM and bufalin = 25.9 μM, respectively) while dehydrobufotenin showed low cytotoxic with high LD_50_ value (235.76 μM) (Table 5).

Evaluating the selectivity index (SI), although compounds marinobufotoxin, marinobufagin and bufalin have shown potentially active, only the compound dehydrobufotenin showed high selectivity for the parasites when analyzed by MTT assay (SI>10) (Table 5).

### ADMET likeness properties

Pharmacokinetic behavior of a compound can determine the success or failure of its biological activity [53]. New potential antimalarial candidates must present good oral bioavailability and good membrane permeability as properties that can lead the *in vivo* experiments to reach success [54]. In this sense, SwissADME web tool [30] allows an *in silico* inference of the main physical-chemical and pharmacokinetic properties of the compounds. In Table 6 are presented the SwissADME profile of the four compounds. 

Bufalin, the most active compound, exhibits numbers of hydrogen bond acceptors (NHA) and hydrogen bond donors (NHD) in accordance with the rule of five by Lipinski. The LogS predicition of bufalin is -5.2, comparable with chloroquine (-6.92), indicating a good solubility. Although, the predicted polar surface area (PSA) of 70.67 Å2 for bufalin suggests that the polarity of this compound is a limiting factor for oral bioavailability [55]. In counterpoint, the synthetic accessibility of bufalin (5.56) is within the range of a non-complicated synthetic accessibility. bufalin could be a potential template for new antimalarial candidates.

**Table 6.** Physicochemical properties of dehydrobufotenin, marinobufotoxin, marinobufagin, bufalin and the antimalarial chloroquine according to SwissADME web tool.

PHYSICOCHEMICAL PROPERTIES	Dehydrobufotenine	Marinobufotoxin	Marinobufagin	Bufalin	Chloroquine
Formula	C12H15N2O	C38H56N4O9	C24H32O5	C24H34O4	C18H26ClN3
Molecular weight	203.26 g/mol	712.87 g/mol	400.51 g/mol	386.52 g/mol	319.87 g/mol
Num. heavy atoms	15	51	29	28	22
Num. arom heavy atoms	9	6	6	6	10
Fractions Csp3	0.33	0.76	0.79	0.79	0.50
Num. rotable bonds	0	18	1	1	8
Num. H-bond acceptors	2	10	5	4	2
Num. H-bond donors	2	6	2	2	1
Molar Refractivity	66.18	190.16	108.86	109.86	97.41
TPSA	36.02 Å²	217.57 Å²	83.20 Å²	70.67 Å²	28.16 Å²
**LIPOPHILICITY**					
Log *P*_olw_ (ILOGP)	-1.27	3.47	3.27	3.34	3.95
Log *P*_olw_ (XLOGP3)	1.64	3.23	2.50	3.2	4.63
Log *P*_olw_ (WLOGP)	1.62	4.10	3.37	4.24	4.62
Log *P*_olw_ (MLOGP)	-2.19	2.12	2.75	3.58	3.20
Log *P*_olw_ (SILICOS-IT)	2.15	4.89	3.82	3.99	4.32
Consensus Log *P* olw	0.39	3.56	3.14	3.67	4.15
**WATER SOLUBILITY**					
Log S (ESOL)	-2.58	-5.19	-3.99	-4.35	-4.55
Solubility	5.38e-01 mg/mL; 2.65e-03 mol/L	4.56e-03 mg/mL; 6.40e-06 mol/L	4.14e-02 mg/mL; 1.03e-04 mol/L	1.75e-02 mg/mL; 4.52e-05 mol/L	9.05e-03 mg/mL; 2.83e-05 mol/L
Class	Soluble	Moderately soluble	Soluble	Moderately soluble	Moderately soluble
Log S (Ali)	-2.01	-7.47	-3.89	-4.36	-4.95
Solubility	1.99e+00 mg/mL; 9.78e-03 mol/L	2.40e-05 mg/mL; 3.37e-08 mol/L	5.13e-02 mg/mL; 1.28e-04 mol/L	1.70e-02 mg/mL; 4.41e-05 mol/L	3.61e-03 mg/mL; 1.13e-05 mol/L
Class	Soluble	Poorly soluble	Soluble	Moderately soluble	Moderately soluble
Log S (SILICOS-IT)	-4.09	-7.46	-4.73	-5.2	-6.92
Solubility	1.63e-02 mg/mL; 8.04e-05 mol/L	2.45e-05 mg/mL; 3.44e-08 mol/L	7.42e-03 mg/mL; 1.85e-05 mol/L	3.68e-03 mg/mL; 9.53e-06 mol/L	3.86e-05 mg/mL; 1.21e-07 mol/L
Class	Moderately soluble	Poorly soluble	Moderately soluble	Moderately soluble	Poorly soluble
**PHARMACOKINETICS**					
GI absorption	High	Low	High	High	High
BBB permeant	Yes	No	No	Yes	Yes
P-gp substrate	Yes	Yes	Yes	Yes	No
CYP1A2 inhibitor	Yes	No	No	No	Yes
CYP2C19 inhibitor	No	No	No	No	No
CYP2C9 inhibitor	No	No	No	No	No
CYP2D6 inhibitor	No	No	Yes	No	Yes
CYP3A4 inhibitor	No	Yes	No	No	Yes
Log Kp (skin permeation)	-6.38 cm/s	-8.36 cm/s	-6.97 cm/s	-6.39 cm/s	-4.96 cm/s
**DRUGLIKENESS**					
Lipinski	Yes; 0 violation	No; 3 violations: MW>500, NorO>10, NHorOH>5	Yes; 0 violation	Yes; 0 violation	Yes; 0 violation
Ghose	Yes	No; 3 violations: MW>500, NorO>10, NHorOH>5	Yes	Yes	Yes
Veber	Yes	No; 2 violations: Rotors>10, TPSA>140	Yes	Yes	Yes
Egan	Yes	No; 1 violation: TPSA>131.6	Yes	Yes	Yes
Muegge	Yes	No; 4 violations: MW>600, TPSA>150, Rotors>15, H-don>5	Yes	Yes	Yes
Biovailability Score	0.55	0.17	0.55	0.55	0.55
**MEDICINAL CHEMISTRY**					
PAINS	0 alert	0 alert	0 alert	0 alert	0 alert
Brenk	1 alert quaternary_nitrogen_2	3 alerts: Three-membered_heterocycle, imine_1, imine_2	1 alert: Three-membered_heterocycle	0 alert	0 alert
Leandlikeness	No; 1 violation: MW<250	No; 2 violations: MW>350, Rotors>7	No; 1 violation: MW>350	No; 1 violation: MW>350	No; 2 violations: Rotors>7, XLOGP3>3.5
Synthetic accessibility	2.14	7.71	6.07	5.56	2.76

## Discussion

The drug discovery process is a major challenge in the pharmaceutical science due the time and money employed in all the phases of developing of a new drug entity [31]. Aiming to reduce cost and time in this process, structure-based virtual screening is an important *in silico* technique for drug design [56].

In this context, BraMMT database with 35 molecular targets of *Plasmodium falciparum* was used in this work. Table 1 shows all the targets and location of the proteins that were used for *in silico* binding assays with compounds isolated from *R. marina*. Three compounds interacted significantly with 10 potential targets (Table 2). 

Of all 35 potential targets of BraMMT, the hexose transporter of *Plasmodium falciparum* (PfHT) interacted significantly with all tested compounds (Table 2). The target PfHT is a membrane protein of the parasite responsible for glucose transport. During the biological development of the parasites in the host´s red blood cells, the plasmodium requires glucose whose uptake is driven by carrier proteins. In red blood cells infected by *P. falciparum* glucose consumption is increased provided by PfHT. Inhibition of glucose transport to infected red blood cells impairs the parasite's metabolism, leading to death. Therefore, compounds that inhibit PfHT can be considered promising in the development of new bioactive compounds capable of treating malaria infections [53]. Additional potential targets analyzed are related to other structures of the parasite, such as apicoplast, cytoplasm, digestive vacuole, and sarcoplasmic reticulum. 

The PfHT protein is a *P. falciparum* hexose transporter. Figures 2, 3, 4, 5 and 6 show the interaction of test compounds with PfHT. It is possible to visualize in the figures the chemical bonds that occurred between the compound and the target. All compounds interact at the same PfHT binding site of *D*-glucose [53] with residues GLN169 and THR145. The compounds interact with the PfHT receptor mainly through Van der Walls interactions, hydrogen bonds, and electrostatic bonds.

Figures 2, 3 and 4 shows that the electrostatic bonds interact with the PfHT receptor by the 2-pyrone group and the perhydrophenanthrene nucleus, demonstrating that the expression of antimalarial activity is associated with the presence of these structures. These groups are common to bufodianolides suggesting that the expression of antimalarial activity is associated with the presence of these structures.

One of the parameters introduced in the rational development of new drugs is Lipinski's rules “rule of five”. These parameters include molecular weight (M.M.) ≤ 500 g/mol, number of hydrogen bonding donor atoms ≤ 5, number of hydrogen bond acceptor atoms ≤ 10, and calculated octanol/water partition coefficient (cLogP) ≤ 5 [42]. The partition coefficient (CLogP) is a measure of the lipophilicity of a substance related to the interaction of the compound with the medium. This is an important tool regarding the study of absorption and transport. Furthermore, the program for the evolution of hazardous compounds recommends this measure, as it also provides estimates of toxicological factors [57].

Gleeson [58] suggests in his study that compounds with CLogP less than 4 and molecular weight less than 400 have a more favorable ADMET profile than those suggested by Lipinski. Following the Gleeson theory, all compounds have CLogP < 4 and compounds dehydrobufotenin, marinobufagin and bufalin have molecular mass below 400. Therefore, these three compounds (dehydrobufotenin, marinobufagin and bufalin) also fit Gleeson's theory. Therefore, compounds dehydrobufotenin, marinobufagin and bufalin showed sufficiently acceptable absorption, distribution, metabolism, excretion, and toxicity properties, according to Lipinski's rule and Gleeson's theory. 

Compounds with high molecular weight and with an excessive number of hydrogen acceptor and donor groups, have greater difficulty in crossing the lipid bilayer of cell membranes. This is because such characteristics increase the lipophilicity of the compound, hindering solubility, and therefore impacting and the drug oral bioavailability [59]. Based in all these definitions, among the four compounds investigated in this study, the dehydrobufotenin was the one that presented the most favorable ADMET properties. 

Secretions from 2 toad species, *R. marina* and *R. guttatus*, were chemically investigated previously. Two extracts and a pure substance (telocinobugagin) presented potential antimalarial activity [18]. When analyzed the IC_50_ values of all compounds tested ensure that IC_50_ values for the tested compounds ranged from 3.44 μM to 19.11 μM (dehydrobufotenin, marinobufotoxin, marinobufagin and bufalin). 

According Mahmoudi [60], a potentially effective antimalarial compound possess an IC_50_ than 10 μM. The results published by Torres [52], indicated that alkaloids isolated from different parts of the *Aspidosperma ulei* plants, were moderately active against *P. falciparum*. These compounds presented IC_50_ values close to 20 μM. Based on this theories, the compounds marinobufotoxin (5.31 μM), marinobufagin and bufalin (3.89 μM and 3.44 μM, respectively) were considered potentially active while dehydrobufotenin (19.11 μM) expresses moderate activity. 

The compounds marinobufotoxin, marinobufagin and bufalin showed cytotoxic activities against human pulmonary fibroblast cells (WI-26VA4) in MTT assay (LD_50_ = 8.89 μM; 3.04 μM and 25.9 μM, respectively). These results corroborate with previous studies that have reported a higher cytotoxic activity of venom extracts from *R. marina* in comparison to those from *R. guttatus* due the presence of 2 other bufadienolides (telocinobufagin, and resibufogenin) [13]. Similarly, extracts of *R. marina* venom from Peruvian Amazon with different compositions showed higher cytotoxic activity in antiproliferative tests with different tumor cell lines [21]. 

In our study the dehydrobufotenin compound showed the highest LD_50_ value (235.76 μM), indicating no cytotoxic effect against human pulmonary fibroblast cells. Low cytotoxicity of the bufadienolides fractions (telocinobufagin) against cancer cell lines (HL-60, SF-295, MDA-MB-435, and HCT-8) was also demonstrated [61]. However, to date, this was the first time that isolated dehydrobufotenine molecule was evaluated in cytotoxic test. 

According to Bézivin [62], values higher than 10 (SI>10) is indicative of high selectivity values, whereas values below 10 (SI<10) are considered as low selectivity. In this study, although compounds 2, 3 and 4 were shown to be potentially active, only compound 1 was selective for the parasite, as it presented a selectivity index value greater than 10 (IS> 200). 

In this work, it was important to assess the cytotoxic activity and evaluate the selectivity index for testing natural compounds with possible antimalarial potential. The exclusive observation of the IC_50_ values would result in wrong conclusions about the antimalarial potential of the compounds. 

## Conclusions

In summary, in docking assay all compounds tested promoted interaction between ligand-receptor with 10 targets of *P. falciparum*. Although *in silico* assays predicted good absorption, permeability, and absence of toxicity for three test compounds, *in vitro* assays demonstrated that only one compound expressed antimalarial activity and absence of cytotoxicity. The compound dehydrobufotenin can serve as a prototype molecule for the development of more active compounds.

### Abbreviations

BraMMT: Brazilian Malaria Molecular Targets; DMSO: dimethyl sulfoxide; IBAMA: Brazilian Institute of Environment and Renewable Natural Resources; SISBIO: System for authorization of collection of biological material.
